# Parliament and the Professions

**Published:** 1920-06-12

**Authors:** 


### Parliament and the Professions.

### Morphia and Opium.

THE PRESIDENT OF THE BOARD OF TRADE, in response to a question by Mr. Gilbert, supplied a statement showing the quantity and value of morphia and morphia salts manufactured in the United Kingdom and exported during 1919. The total export to foreign countries was 302,733 ounces, of a value of £314,948. The two chief countries taking supplies were France (140,873 ounces) and the United States (121,474 ounces); 20,237 ounces were exported to British Possessions, the largest importer being Canada (18,501 ounces).

In reply to a question as to the production in India and exports therefrom of opium, the Secretary of State for India has issued statistics which show that the acreage under poppy cultivation increased from 144,561 acres in 1913-14 to 204,186 in 1916-17, and the production from 24,292 maunds to 32,124 maunds in the same period, one maund equalling 82 lb. Statistics since 1917 are not available.. The exports on private account in the year 1918-19 were 10,467 chests, and on Government account 6,811 chests. With the exception of 2,400 chests on Government account, shipped to the United Kingdom, practically the whole exports were exported to Eastern countries.

### Voluntary Hospitals.

DR. ADDISON informed Mr. Leonard Lyle that it is not intended that the Government should take over voluntary hospitals, but he could not discuss within the limits of a Parliamentary question, the exact relation of voluntary institutions to the general scheme of public health services. The Ministry of Health are giving attention to the question of the financial position of the voluntary hospitals from day to day, and a satisfactory statement is expected to be issued before long.

### Soldiers Still in Hospital.

SIR A. WILLIAMSON stated that there are approximately 5,000 soldiers in hospital in the United Kingdom who are eligible for demobilisation; their daily cost is approximately £2,500.

